# Supplementary material for: WASP family proteins regulate the mobility of the B cell receptor during signaling activation
Source: Nat Commun. 2020 Jan 23;11:439. doi: 10.1038/s41467-020-14335-8 (PMC6978525; doi:10.1038/s41467-020-14335-8)
Supplement: Supplementary file 1 — Supplementary Information [file 41467_2020_14335_MOESM1_ESM.pdf]

Supplementary Figures and Table

Rey-Suarez et al.

WASP family proteins regulate the mobility of the B cell receptor during signaling activation.

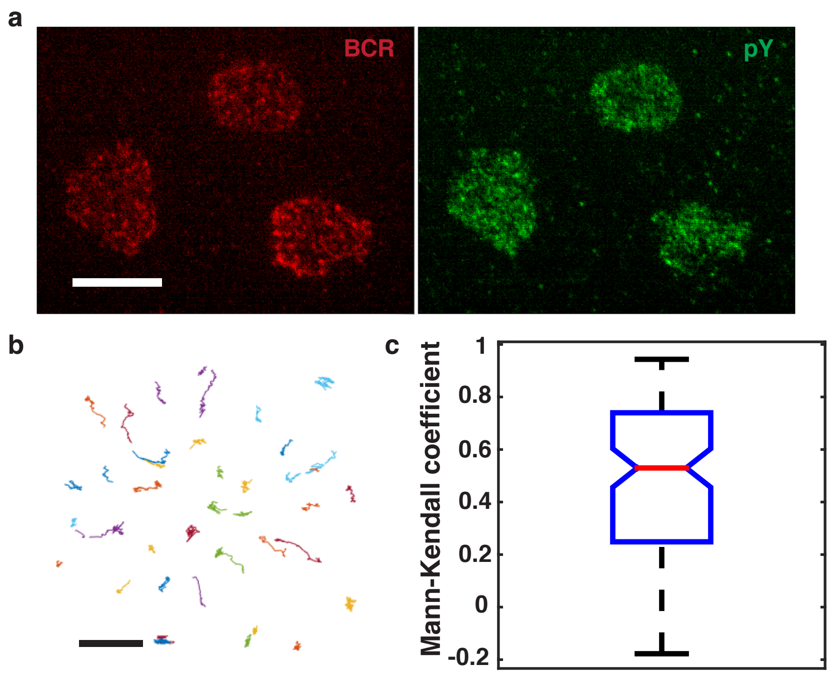

Supplementary Figure 1. B cell signaling activation results in microcluster growth and movement. a) iSIM images of fixed cells (fixed after 3 min of spreading initiation) activated under the same conditions as the single molecule experiments. The red color corresponds to AF546 BCR labeled microclusters while the green is AF488 labeled phosphorylated tyrosine, which also accumulated in clusters. Scale bar is 5 µm. b) BCR microlusters were tracked as they moved towards the center of the cell during the first 200 seconds after the cell contacted the bilayer. Scale bar is 1 µm. c) The intensity within clusters over time was quantified (N = 10 cells) and the Mann-Kendall coefficient was calculated to show the overall increase in intensity over time (MK > 0). The bottom line represents the lower quartile, the upper line represents the upper quartile and the whiskers show the extent of the rest of the data. The red line represents the median.

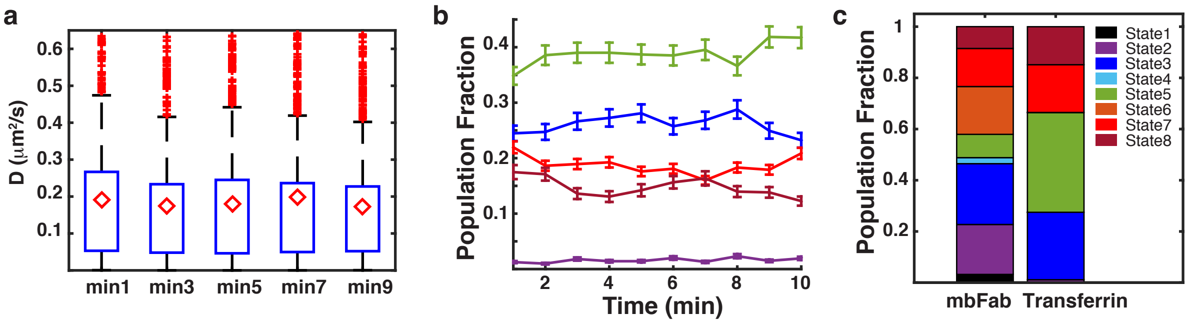

Supplementary Figure 2. Progressive reduction in BCR mobility is specific to BCR activation. a) Boxplot showing BCR diffusivity for cells on a non-activating transferrin coated bilayer measured at 1, 3, 5, 7 and 9 minutes after adding cells. The bottom line represents the lower quartile, the upper line represents the upper quartile, the whiskers show the extent of the rest of the data, and red diamonds represent the mean (N =16 cells). b) Population fractions over time for BCR in cells on non-activating substrate. Error bars represent a confidence interval of 95% on the population fraction calculation. c) Comparative population fractions for BCR in distinct states over the entire time period that cells were imaged for cells on activating (mbFab) and non-activating (transferrin) conditions. Source data are provided as a Source Data file.

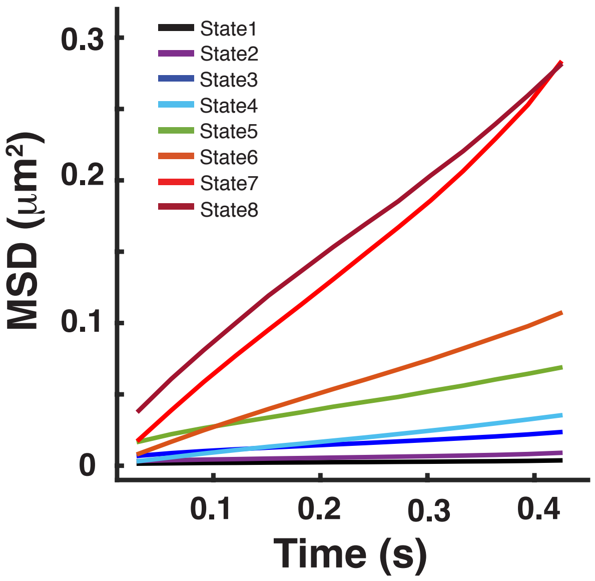

Supplementary Figure 3. Mean square displacement of states for cNKO. Ensemble mean square displacement (eMSD) plots for the 8 diffusive states identified for BCR in cNKO cells. Source data are provided as a Source Data file.

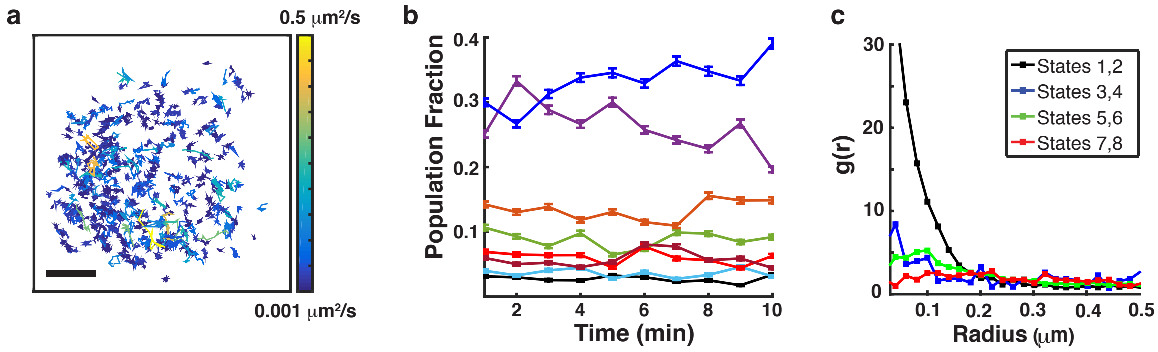

Supplementary Figure 4. Single molecule tracking and pEM analysis of BCR in WASP knockout B cells. a) Tracks of BCR molecules in a WKO cell during a 10 minute period. The tracks are color-coded for diffusivity. b) Plots of population fractions over time for the diffusive states identified in WKO cells. Error bars represent a confidence interval of 95% on the population fraction calculation. c) Plot of pair correlation as a function of distance for all states. Source data are provided as a Source Data file. Data obtained from 30 cells.

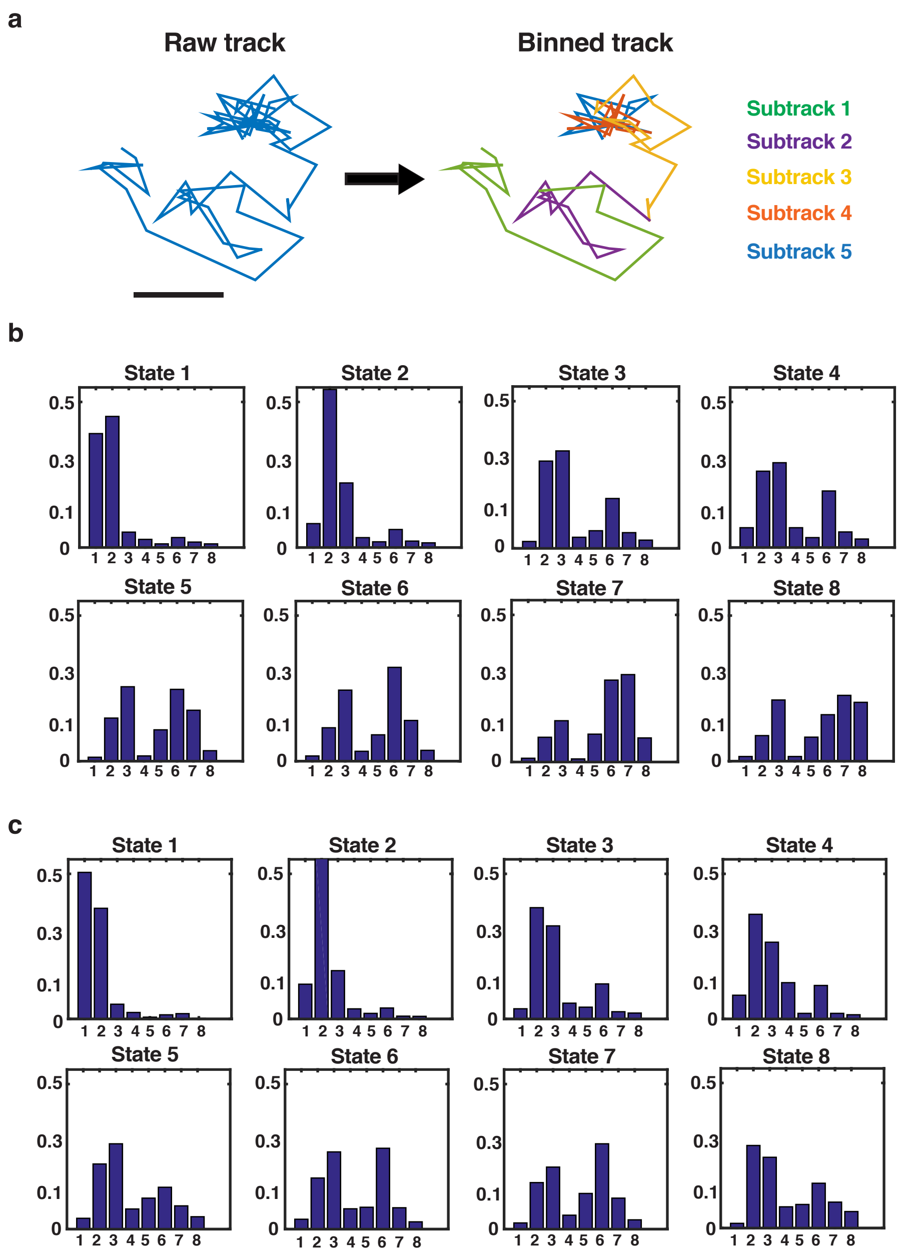

Supplementary Figure 5. Analysis of state transitions for BCR trajectories. a) All single BCR molecule trajectories are split into 15 frame long sub-tracks. pEM analysis is performed over the set of 15 frame long binned tracks and then the original trajectories are reconstructed to obtain information about the transitions of molecules across different states. Scale bar is 1 µm. b) Plots showing the fraction of transitions made from each state to itself and to all others in control cells. Numbers on the x-axis indicate the state to which the transitions are being made. c) Plots showing the fraction of transitions made from each state to itself and to all others in cNKO cells.

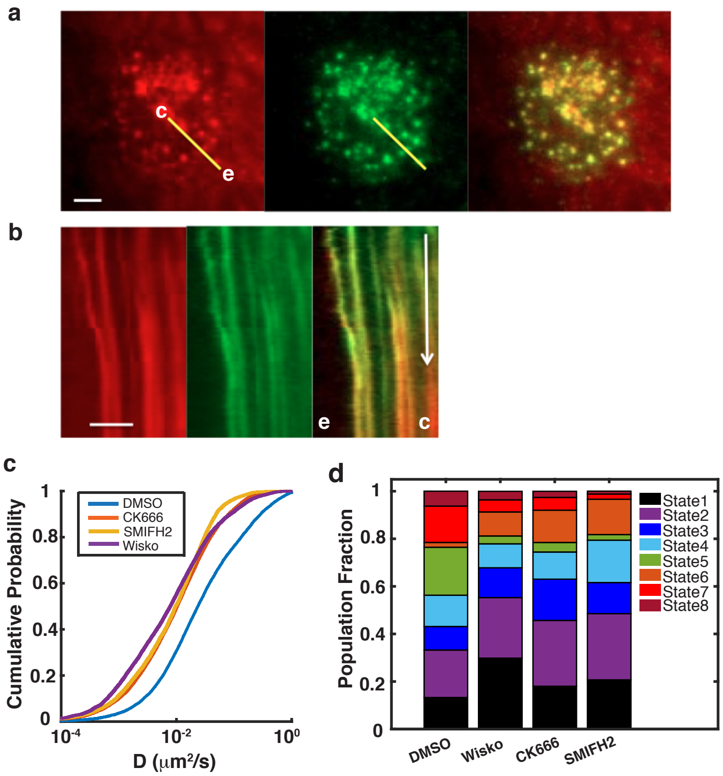

Supplementary Figure 6. Inhibition of actin nucleators affects CD19 diffusivity. a) TIRF images of BCR (red) and CD19 (green) microclusters and the overlay of both channels. b) Kymograph generated along the line indicated in a) showing the inward movement of the microclusters and their superposition as they move. **c** marks the center of the cell and **e** the cell edge. c) Comparison of the cumulative distribution function of CD19 diffusivity in DMSO control cells and cells treated with inhibitors of actin regulatory proteins (CK666, SMIFH2, wiskostatin). ($p < 0.001$, Kruskal-Wallis test for comparison between DMSO and each inhibitor). d) Comparison of population fractions of CD19 diffusivity in control and inhibitor treated cells. (DMSO N=10 cells, CK666 N=11 cells, Wisko N=10 cells, SMIFH2 N=10 cells). Source data are provided as a Source Data file.

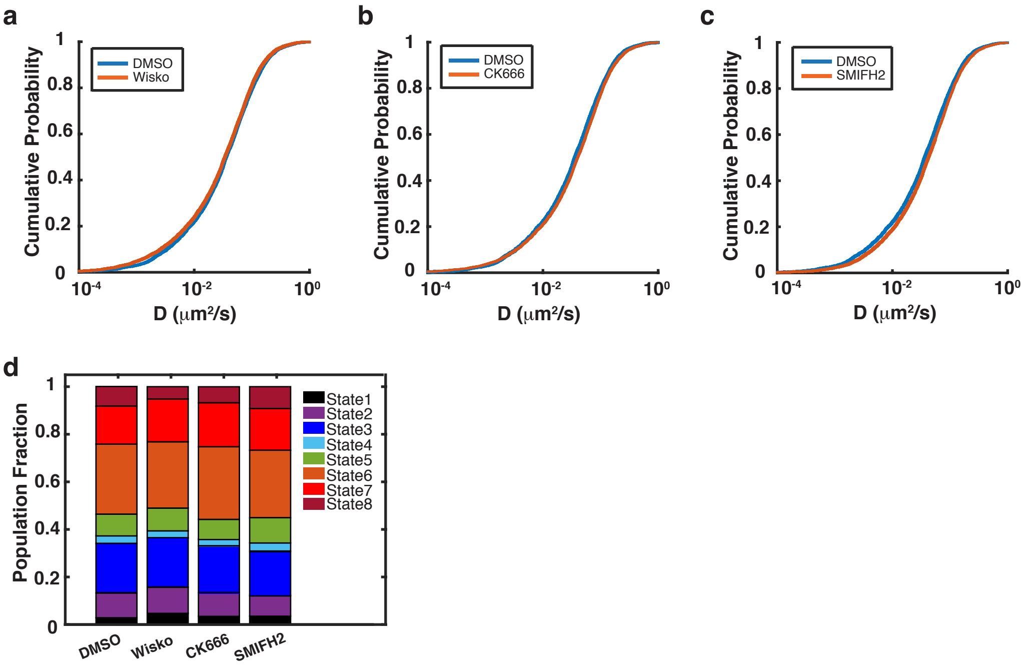

Supplementary Figure 7. Inhibition of actin nucleators does not affect FcγRIIB diffusivity. Cumulative probability plots comparing FcγRIIB diffusivity for control cells with vehicle control DMSO with cells treated with a) Wiskostatin (10 μM), b) CK666 (50 μM) and c) SMIFH2 (25 μM). (Differences were not significant). d) Population fraction comparison of FcγRIIB diffusivity in control and inhibitor treated cells (DMSO N=9 cells, CK666 N=10 cells, Wisko N=8 cells, SMIFH2 N=11 cells).

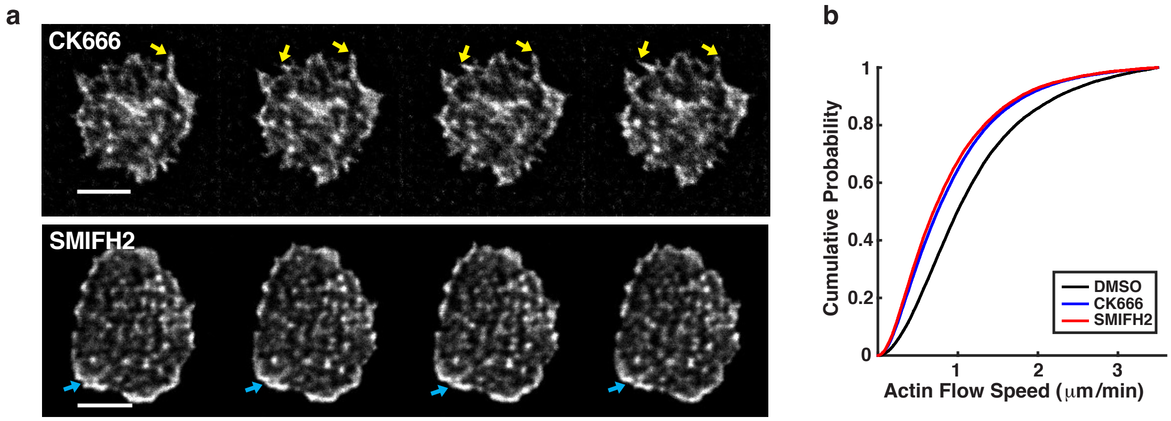

Supplementary Figure 8: Inhibition of actin nucleators reduces actin dynamics. a) iSIM images of activated Lifeact-EGFP B cells at consecutive time points for two conditions: Top: Arp2/3 inhibitor CK666 (50 μM concentration), showing linear actin structures (yellow arrows); Bottom: formin inhibitor SMIFH2 (25 μM concentration), showing loss of actin bundles (blue arrows). The initial time corresponds to 5 min after spreading initiation. Scale bar is 3 μm. b) Cumulative distribution function of actin flow speeds obtained from STICS analysis of 1 minute long movies (at 2 second intervals) for cells treated with DMSO, CK666 and SMIFH2 (N=7 cells for each case). Actin flow speed is significantly different for cells treated with DMSO compared with CK666 and SMIFH2 ($P < 0.0001$ KS test). Data obtained from 7 cells for each condition.

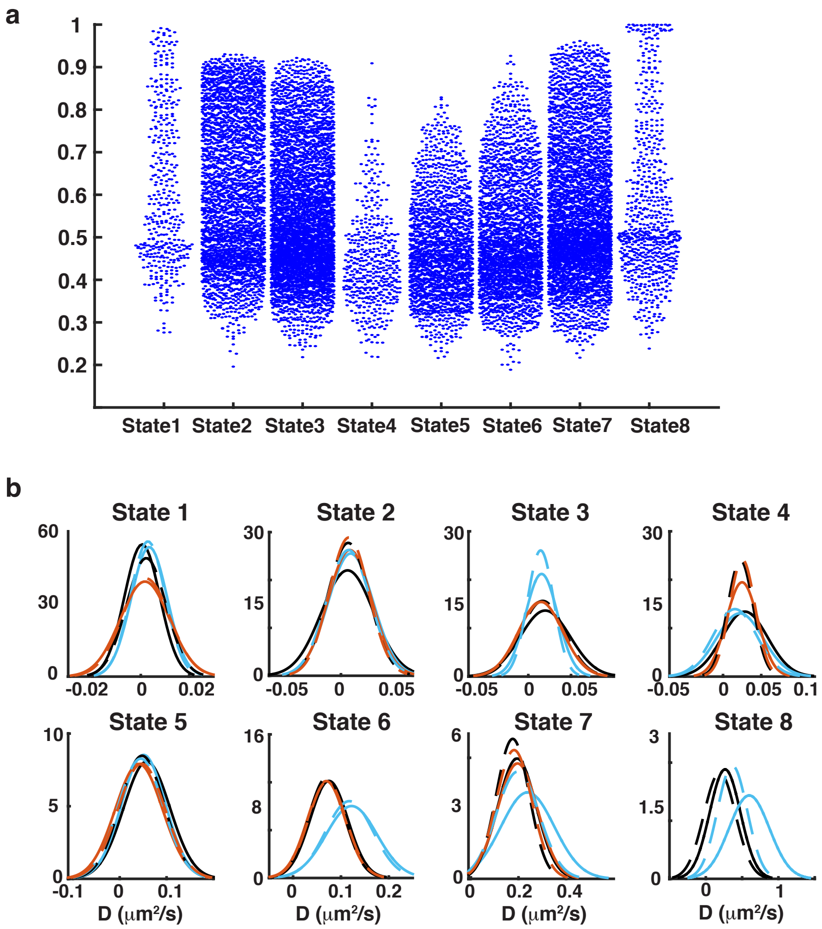

Supplementary Figure 9. Maximum posterior probability and diffusivity distributions for individual states. a) Beeswarm plots showing the maximum posterior probability used to assign a track to a particular state for BCR in control cells. Each point represents a 15 frame long track. b) Plots of diffusivity distributions for each state used to compare across conditions. Black curves correspond to BCR, blue to CD19 and orange to FcγRIIB. The solid lines correspond to control cells and the dashed lines to cNKO cells.

Control v/s cNKO	State 1	State 2	State 3	State 4	State 5	State 6	State 7	State 8
**Percent change**	153.14	70.61	8.46	75.61	-35.72	-31.60	-62.69	-46.99
p	< 0.001	< 0.001	0.01500	0.0324	< 0.001	< 0.001	< 0.001	< 0.001

Control vs WKO	State 1	State 2	State 3	State 4	State 5	State 6	State 7	State 8
**Percent change**	-10.39	35.13	38.28	49.53	-5.23	-27.48	-57.14	-31.27
p	0.02	< 0.001	<0.001	<0.001	0.96	< 0.001	< 0.001	< 0.001

WKO v/s cNKO	State 1	State 2	State 3	State 4	State 5	State 6	State 7	State 8
**Percent change**	182.52	26.25	21.56	17.44	32.16	-5.68	-12.93	-22.87
p	< 0.001	< 0.001	<0.001	<0.001	< 0.001	0.003	< 0.001	< 0.001

Supplementary Table 1. Percentage difference between population fractions for each state across mutants. The percent change is calculated from the difference of the population fraction in the mutants (cNKO, WKO) minus the population fraction from the controls all divided by the control value. The Z test was used to determine the statistical significance of the percentage differences.
