# Supplementary material for: WASP family proteins regulate the mobility of the B cell receptor during signaling activation
Source: Nat Commun. 2020 Jan 23;11:439. doi: 10.1038/s41467-020-14335-8 (PMC6978525; doi:10.1038/s41467-020-14335-8)
Supplement: Supplementary file 3 — Reporting Summary [file 41467_2020_14335_MOESM3_ESM.pdf]

nature research

Corresponding author(s): Arpita Upadhyaya

Last updated by author(s): YYYY-MM-DD

Reporting Summary

Nature Research wishes to improve the reproducibility of the work that we publish. This form provides structure for consistency and transparency in reporting. For further information on Nature Research policies, seeAuthors & Referees and theEditorial Policy Checklist .

Statistics

For all statistical analyses, confirm that the following items are present in the figure legend, table legend, main text, or Methods section.

n/a	Confirmed	
☐	☒	The exact sample size (*n*) for each experimental group/condition, given as a discrete number and unit of measurement
☐	☒	A statement on whether measurements were taken from distinct samples or whether the same sample was measured repeatedly
☐	☒	The statistical test(s) used AND whether they are one- or two-sided<br>*Only common tests should be described solely by name; describe more complex techniques in the Methods section.*
☒	☐	A description of all covariates tested
☐	☒	A description of any assumptions or corrections, such as tests of normality and adjustment for multiple comparisons
☐	☒	A full description of the statistical parameters including central tendency (e.g. means) or other basic estimates (e.g. regression coefficient) AND variation (e.g. standard deviation) or associated estimates of uncertainty (e.g. confidence intervals)
☐	☒	For null hypothesis testing, the test statistic (e.g. *F*, *t*, *r*) with confidence intervals, effect sizes, degrees of freedom and *P* value noted<br>*Give P values as exact values whenever suitable.*
☒	☐	For Bayesian analysis, information on the choice of priors and Markov chain Monte Carlo settings
☒	☐	For hierarchical and complex designs, identification of the appropriate level for tests and full reporting of outcomes
☒	☐	Estimates of effect sizes (e.g. Cohen's *d*, Pearson's *r*), indicating how they were calculated

Our web collection on statistics for biologists contains articles on many of the points above.

Software and code

Policy information about availability of computer code

Data collection	Microscopy data was collected using Andor iQ software (version 3.6.2) and Nikon Elements software (version AR-5.11.02)
Data analysis	All data was analyzed using MATLAB. The code for pEM analysis is freely available at the following link: https://github.com/p-koo/pEMv2

For manuscripts utilizing custom algorithms or software that are central to the research but not yet described in published literature, software must be made available to editors/reviewers. We strongly encourage code deposition in a community repository (e.g. GitHub). See the Nature Research guidelines for submitting code & software for further information.

Data

Policy information about availability of data

All manuscripts must include a data availability statement. This statement should provide the following information, where applicable:

- Accession codes, unique identifiers, or web links for publicly available datasets
- A list of figures that have associated raw data
- A description of any restrictions on data availability

All the data supporting the findings of this study are available from the corresponding author upon reasonable request.

Field-specific reporting

Please select the one below that is the best fit for your research. If you are not sure, read the appropriate sections before making your selection.

☒ Life sciences ☐ Behavioural & social sciences ☐ Ecological, evolutionary & environmental sciences

For a reference copy of the document with all sections, see nature.com/documents/nr-reporting-summary-flat.pdf

Life sciences study design

All studies must disclose on these points even when the disclosure is negative.

Sample size	For receptor diffusivity studies 14 control, 9 cNKO and 3 WKO mice were used. For actin dynamics studies, 3 Lifeact-EGFP mice were used. The number of cells used to collect data for each figure are is indicated in the figure legends. The number of tracks are indicated in the methods.
Data exclusions	No data were excluded from the analyses.
Replication	For each receptor and condition analyzed at least three experiments were performed
Randomization	Randomization was not relevant to our study.
Blinding	Blinding was not relevant to our study. All the single molecule tracks from a given receptor type were pooled together for analysis and state identification was generated by machine learning methods.

Reporting for specific materials, systems and methods

We require information from authors about some types of materials, experimental systems and methods used in many studies. Here, indicate whether each material, system or method listed is relevant to your study. If you are not sure if a list item applies to your research, read the appropriate section before selecting a response.

Materials & experimental systems

n/a	Involved in the study	
☐	☒	Antibodies
☒	☐	Eukaryotic cell lines
☒	☐	Palaeontology
☐	☒	Animals and other organisms
☒	☐	Human research participants
☒	☐	Clinical data

Methods

n/a	Involved in the study	
☒	☐	ChIP-seq
☒	☐	Flow cytometry
☒	☐	MRI-based neuroimaging

Antibodies

Antibodies used	FcγRIIB (CD32) antibody, BD Biosciences, Cat# 553141<br>Alexa Fluor 594 anti mouse CD19 antibody, BioLegend, Cat# 115552, clone 6D5, lot B199758
Validation	Manufacturer's website have provided validation and references

Animals and other organisms

Policy information about studies involving animals; ARRIVE guidelines recommended for reporting animal research

Laboratory animals	Mouse strains C57BL/6 and 129Sv were used to generate knockouts as described in Methods. Mice used for experiments were between 2 to 4 months old with no gender preference.
Wild animals	The study did not involve wild animals.
Field-collected samples	The study did not involve field-collected samples.
Ethics oversight	All experiments involving animals have been approved by the University of Maryland Institution Animal Care and Usage Committee.

Note that full information on the approval of the study protocol must also be provided in the manuscript.
