# Supplementary material for: WASP family proteins regulate the mobility of the B cell receptor during signaling activation
Source: Nat Commun. 2020 Jan 23;11:439. doi: 10.1038/s41467-020-14335-8 (PMC6978525; doi:10.1038/s41467-020-14335-8)
Supplement: Supplementary file 4 — Description of Additional Supplementary Files [file 41467_2020_14335_MOESM4_ESM.pdf]

Description of Additional Supplementary Files

Supplementary Movie 1. Time lapse TIRF images showing the coalescence of clusters and their inward motion in a B cell 2 minutes after spreading initiation on a bilayer. The panels show AF546 labeled mbFab (labeling BCR) in red, AF488 labeled anti-CD19 in green, and the composite of both channels showing the movement and colocalization of clusters.

Supplementary Movie 2. Time lapse images showing actin dynamics in B cells from Lifeact-EGFP mice for DMSO control and Wiskostatin treated cells.
